# Stomach engineering: region-specific characterization of the decellularized porcine stomach

**DOI:** 10.1007/s00383-023-05591-y

**Published:** 2023-11-30

**Authors:** Yusuke Shigeta, Tarek Saleh, Giada Benedetti, Lorenzo Caciolli, Jinke Chang, Elisa Zambaiti, Lei Wu, Sahira Khalaf, Wulei Song, Alessandro Filippo Pellegata, Giovanni Giuseppe Giobbe, Paolo De Coppi

**Affiliations:** 1 Stem Cells and Regenerative Medicine Section, Great Ormond Street Institute of Child Health, University College London, London, UK; 2 Department of Pediatric General and Urogenital Surgery, Juntendo University, Tokyo, Japan; 3 Wellcome / EPSRC Centre for Interventional and Surgical Sciences (WEISS), University College London, London, UK; 4 Centre for Biomaterials in Surgical Reconstruction and Regeneration, Division of Surgery and Interventional Science, University College London, London, UK; 5 Paediatric Surgery, Ospedale Infantile Regina Margherita, Turin, Italy; 6 Laboratory of Biological Structure Mechanics (LaBS), Department of Chemistry, Politecnico di Milano, Milan, Italy; 7 Department of Specialist Neonatal and Paediatric Surgery, Great Ormond Street Hospital, London, UK

**Keywords:** Extracellular matrix, Stomach, Tissue engineering, Decellularization

## Abstract

**Purpose:**

Patients affected by microgastria, severe gastroesophageal reflux, or those who have undergone subtotal gastrectomy, have commonly described reporting dumping syndromes or other symptoms that seriously impair the quality of their life. Gastric tissue engineering may offer an alternative approach to treating these pathologies. Decellularization protocols have great potential to generate novel biomaterials for large gastric defect repair. There is an urgency to define more reliable protocols to foster clinical applications of tissue-engineered decellularized gastric grafts.

**Methods:**

In this work, we investigated the biochemical and mechanical properties of decellularized porcine stomach tissue compared to its native counterpart. Histological and immunofluorescence analyses were performed to screen the quality of decellularized samples. Quantitative analysis was also performed to assess extracellular matrix composition. At last, we investigated the mechanical properties and cytocompatibility of the decellularized tissue compared to the native.

**Results:**

The optimized decellularization protocol produced efficient cell removal, highlighted in the absence of native cellular nuclei. Decellularized scaffolds preserved collagen and elastin contents, with partial loss of sulfated glycosaminoglycans. Decellularized gastric tissue revealed increased elastic modulus and strain at break during mechanical tensile tests, while ultimate tensile strength was significantly reduced. HepG2 cells were seeded on the ECM, revealing matrix cytocompatibility and the ability to support cell proliferation.

**Conclusion:**

Our work reports the successful generation of acellular porcine gastric tissue able to support cell viability and proliferation of human cells.

## Introduction

Patients reporting congenital microgastria [1–4], gastric cancer [5], or post-gastrostomy for gastric rupture repair [6] have been described to present severe gastroesophageal reflux (GER) and dumping syndromes, namely caused by the reduced volume of the stomach. Various surgical techniques have been described as potential treatments [1–3, 5]. However, these therapies have shown little beneficial effects while introducing additional complications for patients [2]. The use of artificial materials for gastric repair has also been described [7–9]. However, these materials have been reported to display inadequate mechanical compliance, plasticity, and ability to accommodate the native anatomy. Moreover, these studies have also described limitations in terms of tissue healing, remodelling, and the ability to accelerate tissue maturation during the growth of the individual.

Decellularization technology is used to produce acellular materials with a low risk of immune rejection upon transplantation [10, 11]. Furthermore, decellularized ECM scaffolds are dynamic, which leads to the continuous extracellular matrix (ECM) deposition and renewal by the recipient’s cells upon migration and repopulation of the transplanted graft. This fact allows the graft to actively accommodate patients’ physiological growth over time, limiting the demand for follow-up surgeries [11]. Decellularized ECM has already been described in several applications. For instance, the literature reports examples of decellularization protocols to derive ECM from intestine [12], trachea [13], heart [10], lungs [14], and liver [15]. Recent works have also shown the potential application of ECM for surgical repair for both human and animal recipients. Singh Rathore et al. reported the use of decellularized buffalo diaphragmatic ECM for abdominal wall defect repair in four different animal models, including buffalo, cow, pig, and goat [11]. Meran et al. recently described the generation of autologous intestinal grafts based on human decellularized intestinal ECM as a proof-of-concept strategy to treat intestinal failure in paediatric patients [16]. Decellularization protocols have also been applied in the field of gastric tissue engineering, as highlighted in the work performed by Zambaiti et al., Feng et al., and Hori et al. [17–19].

The primary goal of gastric tissue engineering is to maintain the generation of functional tissue grafts able to support tissue’s physiological functions (e.g., digestion and nutrient absorption), while limiting the risk of granulation and ulcer formations upon transplantation. At the same time, grafts would also need to accommodate large surface areas, limiting life-threatening pathologies caused by reduced gastric volumes. In this study, we report a method to obtain decellularized gastric tissue from porcine origin. Different anatomical regions of the stomach, including fundus, body, and antrum, were analysed. The decellularized grafts were subjected to biochemical and mechanical characterizations. Furthermore, the decellularized grafts were repopulated with HepG2 cells to investigate their cytocompatibility in vitro.

## Materials and methods

### Collection of organs and decellularization

Whole stomachs were collected from female piglets, weighing approximately 10 kg (obtained from Royal Veterinary College, Hawkshead Campus, UK). After sacrifice, the abdominal wall was disinfected with 70% ethanol (Sigma-Aldrich) in MilliQ water, and a midline incision was performed to expose the abdominal cavity. Then, stomachs were harvested, and their fundus and antrum were connected with Luer-lock connectors (Cole Parmer) using sutures with W792 Mersilk Suture (Size 00, Ethicon). Next, the stomachs were washed with abundant 1X phosphate-buffered saline (PBS; Sigma-Aldrich). After that, stomachs were decellularized by the continuous perfusion of the following reagents: 2% sodium deoxycholate (SDC; Sigma-Aldrich) for 5 h, distilled water (D.W.) overnight, DNase 30 µg/mL (EMD Millipore Corp) in 1X Hank’s Balanced Salt Solution (HBSS; Gibco) for 1 h, 1.5 mol/L sodium chloride in D.W. (NaCl, Sigma-Aldrich) for 30 min, and D.W. with 1% Penicillin/Streptomycin (P /S, Sigma-Aldrich) for 48 h. All the decellularization steps were carried out at room temperature (RT). The perfusion of decellularizing reagents was achieved with an iPump i150 peristaltic pump (iPumps) set to produce a flow rate of 14 mL/min. Decellularized stomachs were stored in 1X PBS with 1% P/S at 4℃. Finally, scaffolds were sterilized via gamma irradiation (3560 Gy).

### Histological staining

Decellularized scaffolds were fixed with 4% paraformaldehyde (PFA, Sigma-Aldrich) for 1 h at RT. After rinsing, they were embedded in optimal cutting temperature embedding medium (OCT; Thermo Scientific). Embedded samples were cryosectioned (Bright Instruments) to produce 7-µm-thick sections for histological staining. Tissue slides were stained with Haematoxylin and Eosin (H&E, Thermo Scientific), Masson’s Trichrome (MT) (RAL Diagnostic), Elastica Van Gieson (EVG, EMD Millipore corp.), and Alcian Blue (AB, Sigma-Aldrich) stains. Native porcine stomach tissue samples from fundus, body, and antrum regions were used as positive controls to ensure that histological stains were correctly performed.

### Immunofluorescence staining

For immunofluorescence (IF) staining, tissue slides were dried and washed with 1X PBS to remove excess OCT. Tissue sections were quenched by incubating them in 50 mM NH_4_Cl (BDH Laboratory) for 1 h at RT. Subsequently, slides were blocked and permeabilized with 0.3% Triton X-100 (Sigma-Aldrich) in PBS (PBS-T) supplemented with 1% bovine serum albumin (BSA, Sigma-Aldrich) for 10 min at room temperature. Primary antibodies were diluted in 0.1% PBS-T supplemented with 1% BSA and applied overnight at 4 °C. Slides were incubated with Alexa Fluor secondary antibodies (Invitrogen) for 1 h at room temperature. Finally, slides were mounted with Anti-Fade Fluorescence Mounting Medium (Abcam). Immunofluorescence images were acquired on a Zeiss LSM710 confocal microscope (ZEISS). Primary antibodies used in this study were anti-Fibronectin (dilution = 1:500, Abcam, ab23751), and anti-Collagen-1 (dilution = 1:500, Novus Biologicals, NB600-450). Secondary antibodies used in this study were AlexaFluor^®^ anti-rabbit 568 (dilution = 1:200, Thermo Fisher, A11011), AlexaFluor^®^ anti-mouse 594 (dilution = 1:200, Thermo Fisher, A11012), and Hoechst 33,342 (Thermo Fisher, H1399) at 10 μg/mL.

### DNA quantification

Tissue fragments, ranging from 15 to 25 mg, were cut from each tissue (decellularized and native porcine stomach). DNA was extracted with DNeasy^®^ Blood & Tissue Kit (QIAGEN), according to the manufacturer’s instructions. DNA concentration was measured with NanoDrop One (Thermo Fisher).

### Glycosaminoglycan quantification

Sulfated glycosaminoglycans content (sGAG) was extracted from native and decellularized tissues (weighing 15–25 mg) using the Blyscan GAG Assay Kit (Biocolor), according to the manufacturer’s instructions. The absorbance was measured at 636 nm using SpectraMax^®^ i3x Multi-Mode Microplate Reader (Molecular devices).

### Soluble collagen quantification

Native and decellularized tissues (weighing 10–20 mg) were used for quantifying the soluble collagen. Sircol^™^ Soluble Collagen Assay Kit (Biocolor) was used according to the manufacturer’s instructions. The absorbance was measured at 556 nm using SpectraMax^®^ i3x Multi-Mode Microplate Reader (Molecular devices).

### Elastin quantification

Elastin content was extracted from native and decellularized tissues (5–10 mg) with Fastin™ Elastin Assay Kit (Biocolor), according to the manufacturer’s instructions. The absorbance was measured at 513 nm with SpectraMax^®^ i3x Multi-Mode Microplate Reader (Molecular devices).

### Mechanical tensile test

Mechanical tensile tests were performed to detect Young’s elastic modulus (MPa), ultimate tensile strength (MPa), and strain at break (%) of decellularized tissues and further highlight the effect of the decellularization process on tissue’s mechanical properties. Instron 5565 tensile testing system (Instron) was used to detect the mechanical parameters. Native and decellularized gastric tissues were cut into 30 mm x 6 mm tissue stripes. Stripes were pinched from both sides so the final working tissue size was 15 mm × 6 mm. The mechanical testing speed was set to 10 mm/min. Mechanical tests were carried out for the stomach’s body regions only.

### HepG2 cell culture

HepG2 cells were cultured on tissue culture plates coated with 0.1% gelatin embryo culture water (Millipore corp.), supplied by Dulbecco’s Modified Eagle’s Medium (DMEM, Gibco supplemented with 10% foetal bovine serum (FBS, Gibco), 1% L-glutamine (Gibco), and 1% Penicillin–Streptomycin (Sigma-Aldrich). HepG2 cells were split 1:3 using TrypPLE reagents (Gibco) and passaged on coated culture plates. HepG2 cells were applied to check the cytocompatibility of the decellularized samples.

### Indirect cytocompatibility

Decellularized scaffolds were processed with a biopsy punch (diameter = 4 mm, Stiefel) to obtain decellularized gastric mucosal discs. They were incubated with HepG2 culture medium at 37 degree for 72 h with agitation. The scaffold-conditioned medium was used to assess the indirect effect of the scaffolds in terms of HepG2 cell viability and proliferation. In particular, the culture medium supernatant (conditioned medium) was further used for the culture of HepG2 cells in gelatinized tissue culture plates as previously described (“Test” condition). A medium containing 20% dimethyl sulphoxide (Sigma-Aldrich)  and an unconditioned medium were used as positive and negative controls, respectively. At 24 h and 96 h of culture, cellular viability was assessed using the live/dead viability/cytotoxicity assay (Molecular Probes, Invitrogen Corp.). This assay utilizes the fluorescent dyes ethidium homodimer and calcein. Ethidium homodimer (red) increases in fluorescence intensity upon binding to DNA. Calcein (green) is hydrolysed by intracellular hydrolases found in living cells and subsequently undergoes an increase in fluorescence intensity. Therefore, in this assay, viable cells fluoresce green while non-viable cells fluoresce red. Live/dead assay was performed as per the manufacturer’s instructions. Imaging was carried out with Axio Observer A1 (Zeiss) and images were further processed with ImageJ (Version Windows 32-bit) software to count the percentage of live/dead cells. The 1-(4,5-dimethylthiazol-2-yl)-3,5-diphenylformazan (MTT) assay (Sigma-Aldrich) was also used to assess cellular proliferation via quantification of the enzyme mitochondrial dehydrogenase. MTT assay was performed according to the manufacturer’s instructions. The absorbance at 570 nm was measured by the SpectraMax^®^ i3x Multi-Mode Microplate Reader (Molecular devices).

###  Direct cytocompatibility

The decellularized gastric mucosal discs were top-seeded with 20 µL of HepG2 cells (1.5 × 105). Repopulated gastric discs were cultured in tissue culture plates supplied with HepG2 culture medium for 72 h at 37, 5% CO_2_. They were then stained by H&E staining to check the cellular attachment. Cellular proliferation was also confirmed by performing immunofluorescence staining for the Ki67 marker (dilution 1: 200, Abcam, ab15580).

### Statistical methods

For three parameters, a one-way ANOVA was performed using GraphPad Prism 9 (National Institutes of Health, V1.52). In the case of two parameters, a T-test was performed with GraphPad Prism 9. In all the presented figures, statistical significance is expressed as ***p < 0.0001, **p < 0.01, *p < 0.05. Quantitative results are expressed as mean ± standard deviation (SD).

## Results

### Histological analysis shows ECM preservation in decellularized tissues from the fundus, body, and antrum

**Fig. 1:**
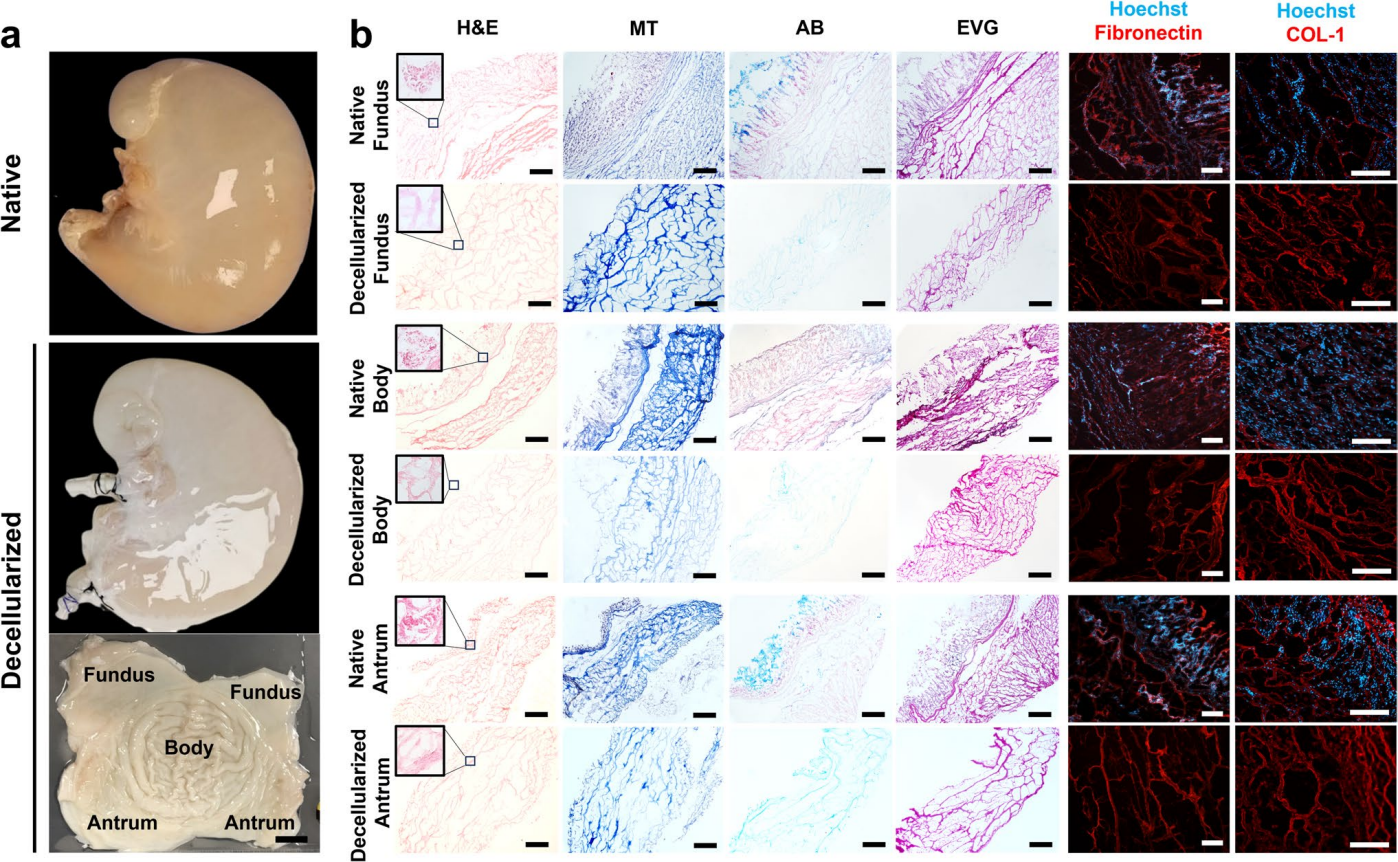
**a** Macroscopic appearance of decellularized porcine stomachs showing preservation of the macroscopic structure compared to the native stomach. The bottom panel showing the opened decellularized stomach, with the different regions annotated. Scale bar = 2 cm. **b** Characterization by histology and immunofluorescence for each location. Haematoxylin and eosin (H&E), Masson’s Trichrome (MT), Elastica Van Gieson (EVG) and Alcian Blue (AB) staining, and immunostaining against fibronectin and collagen I (in red) in decellularized porcine stomachs compared to native stomachs. Scale bar 1000 μm for histologies. Scale bars 100 µm for immunostaining

The macroscopic appearance of the whole organ before and after decellularization was comparable in shape (Fig. 1a), suggesting that the technique did not disrupt the macrostructure of the organ. Nonetheless, the decellularized organs were whitened compared to the native, as a consequence of the removal of all cellular components (Fig. 1a). Moreover, the opening of the decellularized stomach sac displayed an intact inner layer, as no obvious damage was visible (Fig. 1a).

After stomach dissection into its three regions (fundus, body, antrum), histological analysis was performed to compare native and decellularized tissue. H&E staining in the native samples showed the layered structure of the stomach (Fig. 1b). The decellularized samples showed no detectable nuclei in all regions, and the overall ECM ultrastructure was maintained (Fig. 1b). The collagen fibres were identified by MT staining. At all regions, the collagen fibres were preserved in the decellularized samples compared to the native tissue (Fig. 1b). The AB staining was used to detect acidic polysaccharides, such as glycosaminoglycans. In the native tissue, there was the evident presence of pink nuclei marking the cells, mucous cells were identified by light blue cytoplasm, and the glycocalyx was evident on top of the gastric glands layer (Fig. 1b). In the decellularized tissue, only a fade light blue ECM structure was persistent, proving once again the removal of cells, but also the decrease in GAGs composition. EVG staining was used to confirm their retention in the decellularized tissues (Fig. 1b).

The different regions of native or decellularized samples were also analysed by immunofluorescence staining for ECM components (fibronectin and collagen I), with cell nuclei counterstained with Hoechst. In the native tissues, the stomach wall layering was evident, with a clear gland ultrastructure defined by the nuclei (Fig. 1b). No signal marking the nuclei was detected in the decellularized tissue, whereas the fibronectin was kept intact (Fig. 1b). Additionally, collagen I fibres were preserved in the decellularized tissue compared to the native (Fig. 1b).

These macroscopic and histological appearances indicated that decellularization treatment could remove cells successfully while preserving ECM microstructure.

### ECM characterization in decellularized tissues from the fundus, body, and antrum

**Fig. 2:**
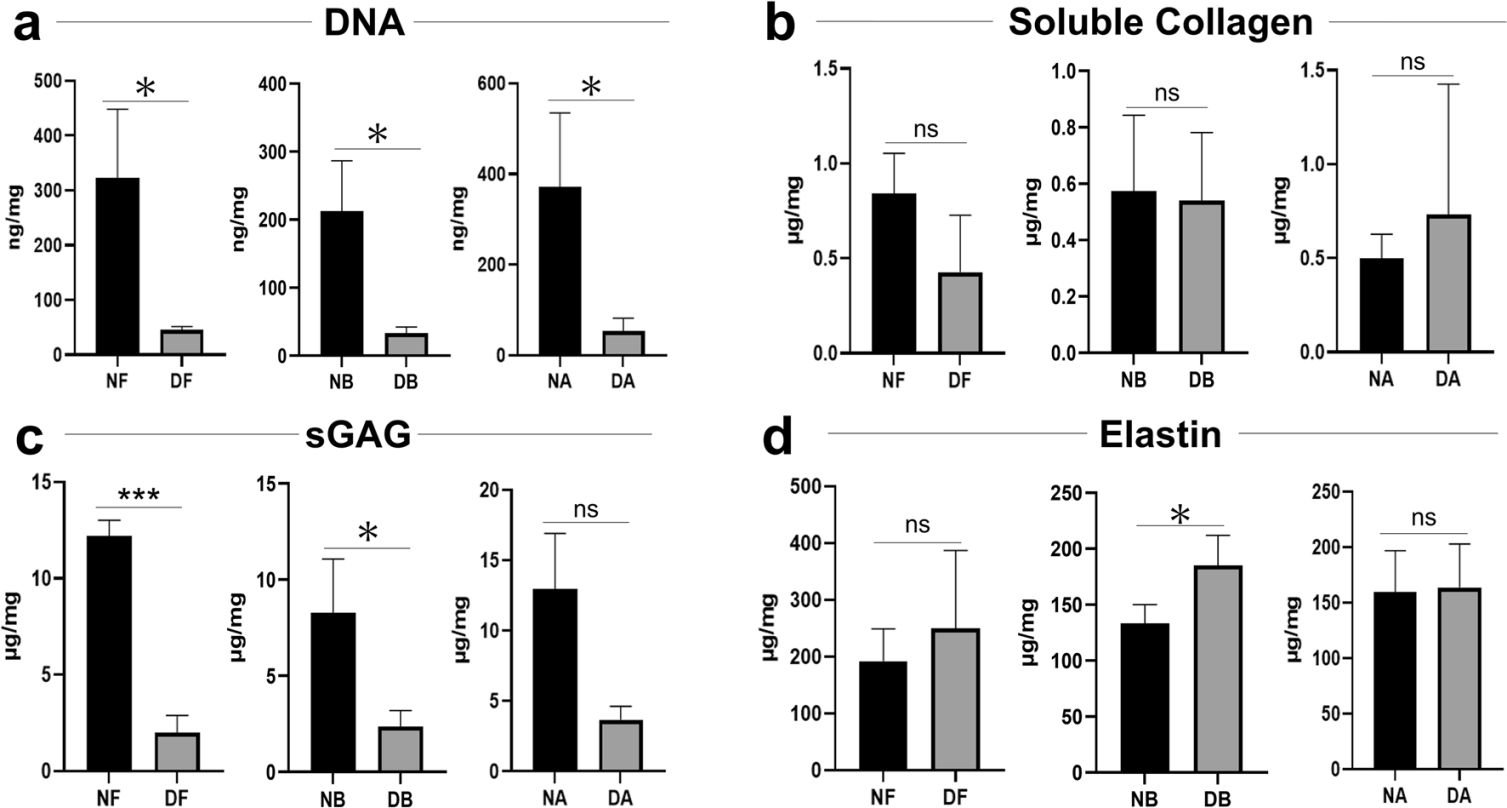
**a** DNA quantification in native and decellularized tissue in the fundus, body, and antrum (*p* < 0.05). **b** Soluble collagen quantification in native and decellularized tissue in the fundus, body, and antrum.** c** Sulfated glycosaminoglycan (sGAG) in native and decellularized tissue in the fundus, body, and antrum (fundus *p* < 0.001: body *p* < 0.05). **d** Elastin quantification in native and decellularized tissue in the fundus, body, and antrum (body *p* < 0.05). NF native fundus, DF decellularized fundus, NB native body, DB decellularized body, NA native antrum, DB decellularized antrum

To further assess the changes in ECM composition, specific assays were performed to quantify the differences between native and decellularized tissue. First, DNA quantification was performed to measure the residual nuclear contents left in the decellularized scaffold. The DNA amount in the decellularized tissue was significantly lower compared to the native tissue at each location (*p* < 0.05) (Fig. 2a).

Coherently with the histology results, quantification of soluble collagen showed no significant difference between native and decellularized tissues for each location (Fig. 2b). There was a significant reduction of sGAG in the decellularized tissue compared to the native tissue. In particular, the difference was statistically significant in the fundus (*p* < 0.0001) and in the body (*p* < 0.05), while a non-significant reduction was present at the antrum (Fig. 2c). Quantification of elastin showed no significant differences at the fundus/antrum between native and decellularized tissues, while it exhibited significantly higher level in decellularized body tissue (*p* < 0.05) (Fig. 2d).

### Mechanical tensile assays

**Fig. 3:**
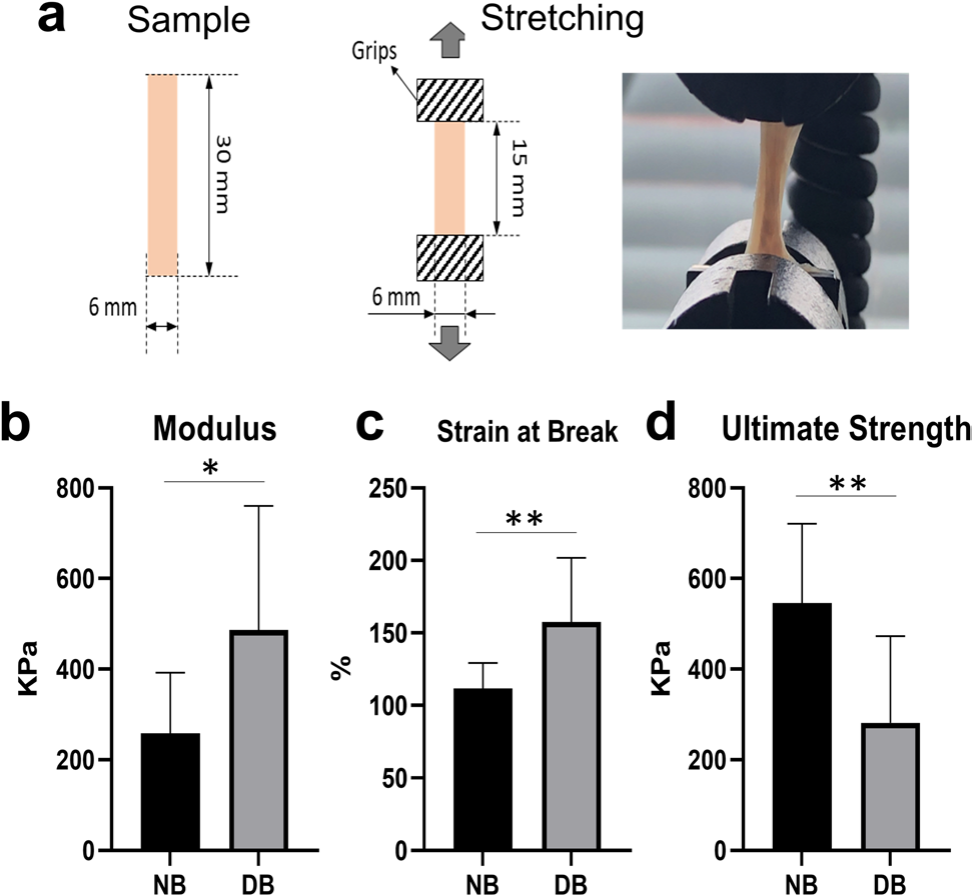
**a** Tissue sample size is 30 mm x 6 mm. The samples are stretched by pinches to both directions. **b**–**d** Tensile test between native and decellularized body tissue in (**b**) Modulus (*p* < 0.05), (**c**) Strain at Break (*p* < 0.005), and (**d**) Ultimate Tensile Strength (*p* < 0.005). NB native body, DB decellularized body

To evaluate the mechanical properties of the decellularized tissue compared to the native, 30-mm-long stripes of gastric body tissue were cut and pinched by the tensile testing machine, leaving a final working tissue size of 15 mm (Fig. 3a). The tensile test showed a significant increase in stiffness in the decellularized tissue (*p* < 0.05), as shown by the Young’s modulus (Fig. 3b). The strain at break (displayed as stretched length rate when the tissue was broken) showed a significant increase in the decellularized tissue (*p* < 0.005) (Fig. 3c), while the ultimate tensile strength (the force required to break the material) was significantly reduced in the decellularized tissue (*p* < 0.005) (Fig. 3d).

### The decellularized tissue is cytocompatible

**Fig. 4:**
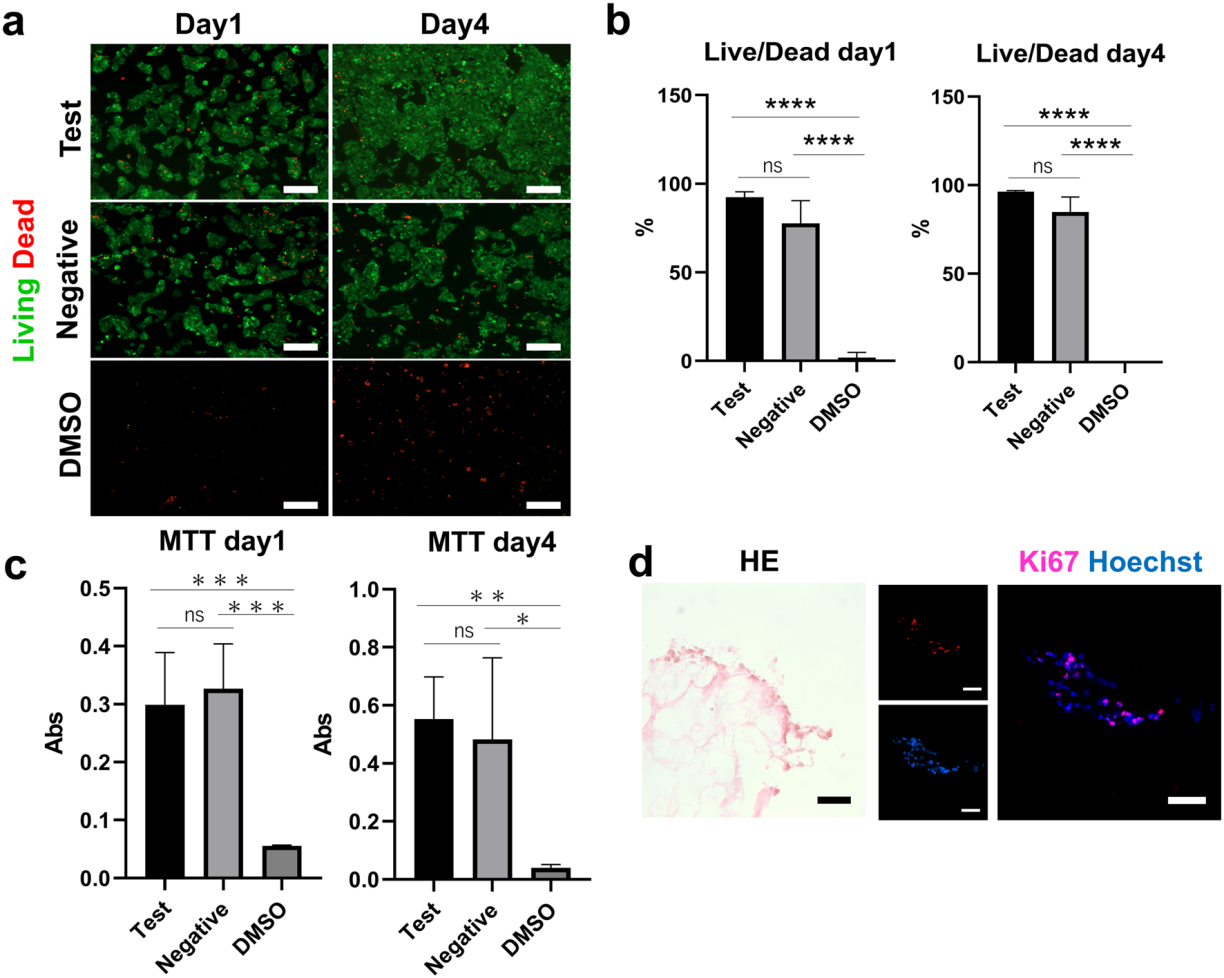
**a**, **b** Live/Dead assay showing the percentage of live cells between test medium and negative control medium for each period (day 1, day 4) and DMSO medium. Scale bar = 400 µm (*p* < 0.0005). **c** MTT assay showing the proliferation levels in test medium and negative control medium for each period (day 1, day 4), and DMSO medium (*p* < 0.001 for day 1, *p* < 0.05 for day 4). **d** H&E and immunostaining with Ki67 staining of HepG2 cells seeded on decellularized scaffold sections. Scale bar = 50 µm

After culturing HepG2 cells for 1 day and 4 days with the different conditioned media, Live/Dead assay and MTT assay were performed. Test and negative groups looked comparable in the Live/Dead assay, while the DMSO group (positive control) clearly showed increased percentage of dead cells (Fig. 4a). Our observation was confirmed upon quantification and there were no significant differences between the test and negative control groups at the different time points in terms of the percentage of live cells (Fig. 4b). Similarly, the MTT assay showed that there was no significant difference between the test and negative groups on either day 1 or day 4, (Fig. 4c). To further test the cytocompatibility of the scaffold, cells were directly seeded on top of it and allowed to grow for 72 h. H&E-stained sections showed the presence of a monolayer of cells over the scaffold patch. The sections were also stained for the proliferation marker Ki67, showing that cells were alive and proliferating also when cultured on the scaffold (Fig. 4d).

## Discussion and conclusion

The stomach is a vital organ responsible for the accumulation of food, hormone secretion, chemical and mechanical digestion, and absorption of some nutrients [20].

Pathological conditions that result in gastric volume reduction, such as microgastria and other disorders that arise consequently to gastrectomy for gastric cancer or gastric perforation, lead to various complications and a decreased quality of life. Therefore, several suggestions for surgery improvement and other procedures have been proposed [1–3]. Although promising, those surgeries are quite invasive for children, and shunting surgery alone does not solve the problem of gastric volume improvement, nor does it compensate for the complex role of signal coordination as earlier described. Therefore, regenerative medicine research is essential.

Gastric regenerative medicine research focuses on the use of tissue engineering to expand stomach volume, with a special focus on tissue transplantation into gastric wall defects [19]. Based on the literature, collagen sponge scaffolds derived from porcine skin, reinforced with a felt derived from polyglycolic acid, were transplanted into the stomach of a dog [19]. The inner surface was covered by the gastric epithelium, which would have been a good result at the time. However, the experiment required silicone sheet removal, increasing the number of procedures required to fulfil the aim. After this, several experiments have been conducted with various materials and cells [21, 22]. An innovative approach was investigated by Ueno et al. by transplanting a porcine small intestinal submucosal sheet (SIS) into rats. The gastric mucosa covered the inner surface of the stomach, showing signs of revascularization. When mesenchymal stem cells (MSCs) were seeded on SIS, smooth muscle was also observed [23]. In a rat-rat transplantation experiment conducted by Maemura et al., neonatal rat gastric epithelial organoids were cultured in a biodegradable polymer and transplanted into the omentum. As a result, a cystic structure with gastric epithelium was obtained, which was then used to replace the stomach of recipient rats. The transplanted rats showed no nutritional differences compared to controls [24]. Moreover, G cells and parietal cells were identified. However, the new stomach obtained by this method was smaller than the stomach of a neonatal rat [24]; hence, further research is needed to develop a technique that can compensate for a larger area while still providing excellent physiological function.

Unlike the oesophagus, none of the published gastric studies have yet been clinically tested in humans [25]. A possible reason for this is the need for larger grafts to be of significance in clinical practice. At present, the largest sizes used in animal models are 4 cm × 4 cm by Hori et al. [19, 26], and 5 cm by Araki et al. [27], and these sizes do not meet the demand. Moreover, the grafts will have to functionally reproduce the original gastric functions, such as migration and engraftment of stable recipient-derived cells, peristalsis by muscle tissue, and absorption.

To answer this unmet need, we have developed an SDC/DNase-based decellularization protocol for the porcine stomach. Our aim is to reproduce the physiological functions of the human stomach by engrafting human cells onto the physiological ECM components of the decellularized porcine stomach. This will ultimately allow the production of larger grafts while reducing the risk of immune rejection, viral and bacterial infections, etc. Our study proposed the characterization of the ECM in the three main regions of the stomach: fundus, body, and antrum.

First, the procedure maintained an intact macrostructure with no damage on the inner layer. Histological analysis and immunostaining proved the efficiency of the decellularization protocol adopted, as cells were successfully removed, while maintaining the ECM components. Further quantifications identified no significant difference in the amount of soluble collagen between native and decellularized tissue in all three regions. Similarly, no significant difference for the same comparison was visible for elastin quantification, except for the body region, where the elastin significantly increased in the decellularized tissue. On the other hand, a decrease in sGAG was found in the decellularized samples, consistently with the AB staining.

The absence of differences in the ECM composition in the three regions could suggest the possibility of using body decellularized tissue (the largest region, therefore the easiest scaffold to be produced) as main source for large grafts production to be used at any region. Therefore, we tested the mechanical properties and cytocompatibility of body decellularized tissue.

Regarding the mechanical test, Young’s elastic modulus and strain at break of the decellularized body were significantly higher compared to the native tissue, while the ultimate tensile strength was significantly lower. The increase of the modulus accounts for the elastic properties of the tissue. The changes in strain at break and UTS point at the ability of the material to increase deformation at lower pressures. Although it was difficult to strictly standardize the orientation of the muscle fibres in the tensile test, we can still indicate the decellularization process to be predominantly responsible for the difference in tissue strength. It is unclear how these differences would affect the outcome of transplantation: the increased elasticity suggests that it would be best to avoid using tissue smaller than the defect at the time of transplantation. Of note, it would be interesting to investigate whether in vivo degradation by recipient-derived cells and ECM remodelling after transplantation could revert elasticity to physiological tissue strength.

Finally, we showed that HepG2 cells (human-derived cells) could be seeded on porcine stomach-derived ECM or grown in an ECM-conditioned medium. They were able to grow and proliferate, proving the produced ECM scaffold is cytocompatible. To further improve the complexity of the graft for transplantation, it will be necessary to use cell types such as gastric epithelial cells, fibroblasts, myocytes, endothelial cells, and enteric nervous system cells. Indeed, the use of HepG2 cells, which is a cell line derived from a human tumour, could bias our observation on the cellular proliferation rate, which could be higher than gastric epithelial cells. Therefore, the use of gastric epithelial organoids as a cell source for seeding the ECM patch could be an interesting and reliable solution.

Overall, we could reliably produce decellularized porcine patches from the three main gastric regions. The absence of differences in ECM composition among the different regions allowed us to identify the body as the main source of scaffold, allowing the production of large-size patches. It is important to note that our ECM did not show any toxicity effect on human cells, which grew on decellularized porcine tissue. These results introduce this system as a potential candidate for future animal transplantation studies.

## Data Availability

The authors declare that all data supporting the findings of this study are available within the article, main figures and figure legends, or from the authors upon reasonable request.

## References

[1] Hattori K, Bvulani B, Numanoglu A, Cox S, Millar A (2016) Total esophageal gastric dissociation for the failed antireflux procedure in a child with microgastria. Eur J Pediatr Surg Rep 4(1):006–009. 10.1055/S-0035-1571176 10.1055/s-0035-1571176 PMC5177559 28018800

[2] Ruczynski LIA, Botden SMBI, Daniels-Scharbatke HE, Schurink M, De Blaauw I (2021) Treatment of congenital microgastria. Eur J Pediatr Surg 31(2):129–134. 10.1055/S-0040-1710027 32422678 10.1055/s-0040-1710027

[3] Filisetti C, Maestri L, Meroni M, Marinoni F, Riccipetitoni G (2017) Severe dumping syndrome in a 6-year-old girl with congenital microgastria treated by Hunt–Lawrence pouch. Eur J Pediatr Surg Rep 5(1):e17. 10.1055/S-0037-1601305 10.1055/s-0037-1601305 PMC5371510 28361011

[4] Furman MS, Connolly SA, Brown SD, Callahan MJ (2021) The pediatric stomach-congenital abnormalities. Pediatr Radiol 51(13):2461–2469. 10.1007/S00247-021-05155-Z 34351495 10.1007/s00247-021-05155-z

[5] Heimbucher J et al (1994) Motility in the Hunt–Lawrence pouch after total gastrectomy. Am J Surg 168(6):622–626. 10.1016/S0002-9610(05)80133-3 7978007 10.1016/s0002-9610(05)80133-3

[6] Lin CM et al (2008) Neonatal gastric perforation: report of 15 cases and review of the literature. Pediatr Neonatol 49(3):65–70. 10.1016/S1875-9572(08)60015-7 18947001 10.1016/S1875-9572(08)60015-7

[7] Ono H, Takizawa K, Kakushima N, Tanaka M, Kawata N (2015) Application of polyglycolic acid sheets for delayed perforation after endoscopic submucosal dissection of early gastric cancer. Endoscopy 47(Suppl 1 UCTN):E18–E19. 10.1055/S-0034-1390730 25603508 10.1055/s-0034-1390730

[8] Takimoto K, Imai Y, Matsuyama K (2014) Endoscopic tissue shielding method with polyglycolic acid sheets and fibrin glue to prevent delayed perforation after duodenal endoscopic submucosal dissection. Dig Endosc 26(Suppl 2):46–49. 10.1111/DEN.12280 24750148 10.1111/den.12280

[9] Tsujii Y et al (2015) Polyglycolic acid sheets for repair of refractory esophageal fistula. Endoscopy 47(Suppl 1):E39–E40. 10.1055/S-0034-1390914 25603520 10.1055/s-0034-1390914

[10] Ashfaq A, Brown T, Reemtsen B (2017) Repair of complete atrioventricular septal defects with decellularized extracellular matrix: initial and midterm outcomes. World J Pediatr Congenit Heart Surg 8(3):310–314. 10.1177/2150135116684797 28520544 10.1177/2150135116684797

[11] Singh Rathore H et al (2018) Use of the bubaline acellular diaphragm matrix (ADM) for repair of abdominal wall defects in four different species of animals. Int J Chem Stud 6(4):157–161

[12] Giobbe GG et al (2019) Extracellular matrix hydrogel derived from decellularized tissues enables endodermal organoid culture. Nat Commun. 10.1038/s41467-019-13605-4 31827102 10.1038/s41467-019-13605-4 PMC6906306

[13] Hamilton NJ et al (2015) Tissue-engineered tracheal replacement in a child: a 4-year follow-up study. Am J Transplant 15(10):2750–2757. 10.1111/AJT.13318 26037782 10.1111/ajt.13318 PMC4737133

[14] Farré R, Otero J, Almendros I, Navajas D (2018) Bioengineered lungs: a challenge and an opportunity. Arch Bronconeumol 54(1):31–38. 10.1016/J.ARBRES.2017.09.002 29102342 10.1016/j.arbres.2017.09.002

[15] Mazza G et al (2015) Decellularized human liver as a natural 3D-scaffold for liver bioengineering and transplantation. Sci Rep. 10.1038/SREP13079 26248878 10.1038/srep13079 PMC4528226

[16] Meran L et al (2020) Engineering transplantable jejunal mucosal grafts using patient-derived organoids from children with intestinal failure. Nat Med 26(10):1593–1601. 10.1038/s41591-020-1024-z 32895569 10.1038/s41591-020-1024-z PMC7116539

[17] Zambaiti E et al (2019) Whole rat stomach decellularisation using a detergent-enzymatic protocol. Pediatr Surg Int 35(1):21–27. 10.1007/S00383-018-4372-8 30443739 10.1007/s00383-018-4372-8 PMC6326006

[18] Feng L et al (2021) Decellularized gastric matrix as a mesh for gastric perforation repair. J Biomed Mater Res B Appl Biomater 109(3):451–462. 10.1002/JBM.B.34713 32841467 10.1002/jbm.b.34713

[19] Hori Y, Nakamura T, Matsumoto K, Kurokawa Y, Satomi S, Shimizu Y (2001) Experimental study on in situ tissue engineering of the stomach by an acellular collagen sponge scaffold graft. ASAIO J 47(3):206–210. 10.1097/00002480-200105000-00008 11374758 10.1097/00002480-200105000-00008

[20] Soybel DI (2005) Anatomy and physiology of the stomach. Surg Clin North Am 85(5):875–894. 10.1016/J.SUC.2005.05.009 16139026 10.1016/j.suc.2005.05.009

[21] Sîrbu-Boeţi MP, Chivu M, Pâslaru LL, Efrimescu C, Herlea V, Pecheanu C, Moldovan L, Dragomir L, Bleotu C, Ciucur E, Vidulescu C, Vasilescu M, Boicea A, Mănoiu S, Ionescu MI, Popescu I (2009) “Transplantation of mesenchymal stem cells cultured on biomatrix support induces repairing of digestive tract defects, in animal model. Chirurgia (Bucur) 104(1):55–65 (**PMID: 19388570**) 19388570

[22] Sala FG, Kunisaki SM, Ochoa ER, Vacanti J, Grikscheit TC (2009) Tissue-engineered small intestine and stomach form from autologous tissue in a preclinical large animal model. J Surg Res 156(2):205–212. 10.1016/J.JSS.2009.03.062 19665143 10.1016/j.jss.2009.03.062

[23] Ueno T et al (2007) Functional evaluation of the grafted wall with porcine-derived small intestinal submucosa (SIS) to a stomach defect in rats. Surgery 142(3):376–383. 10.1016/J.SURG.2007.04.019 17723890 10.1016/j.surg.2007.04.019

[24] Maemura T et al (2008) A tissue-engineered stomach shows presence of proton pump and G-cells in a rat model, resulting in improved anemia following total gastrectomy. Artif Organs 32(3):234–239. 10.1111/J.1525-1594.2007.00528.X 18201286 10.1111/j.1525-1594.2007.00528.x

[25] Nieponice A et al (2014) Patch esophagoplasty: esophageal reconstruction using biologic scaffolds. Ann Thorac Surg 97(1):283–288. 10.1016/J.ATHORACSUR.2013.08.011 24266951 10.1016/j.athoracsur.2013.08.011

[26] Hori Y et al (2002) Functional analysis of the tissue-engineered stomach wall. Artif Organs 26(10):868–872. 10.1046/J.1525-1594.2002.07006.X 12296927 10.1046/j.1525-1594.2002.07006.x

[27] Araki M et al (2009) Development of a new tissue-engineered sheet for reconstruction of the stomach. Artif Organs 33(10):818–826. 10.1111/J.1525-1594.2009.00808.X 19839991 10.1111/j.1525-1594.2009.00808.x

